# Promoting Developmental Potential in Early Childhood: A Global Framework for Health and Education

**DOI:** 10.3390/ijerph18042007

**Published:** 2021-02-19

**Authors:** Verónica Schiariti, Rune J. Simeonsson, Karen Hall

**Affiliations:** 1 Division of Medical Sciences, University of Victoria, Victoria, BC V8W 2Y2, Canada; 2 School Psychology Program, School of Education, University of North Carolina, Chapel Hill, NC 27599, USA; rjsimeon@email.unc.edu (R.J.S.); karench@live.unc.edu (K.H.); 3 School of Education and Communication, Jönköping University, SE-551 11 Jönköping, Sweden

**Keywords:** early child development, ICF, education, health, inequality, rights, functioning, potential, intervention, disability

## Abstract

In the early years of life, children’s interactions with the physical and social environment- including families, schools and communities—play a defining role in developmental trajectories with long-term implications for their health, well-being and earning potential as they become adults. Importantly, failing to reach their developmental potential contributes to global cycles of poverty, inequality, and social exclusion. Guided by a rights-based approach, this narrative review synthesizes selected studies and global initiatives promoting early child development and proposes a universal intervention framework of child-environment interactions to optimize children’s developmental functioning and trajectories.

## 1. Introduction

Access to health and education services, are essential factors for optimal development, functioning and well-being of the child, factors paralleling the development, functioning and well-being of family, community, and society. Limited or deprived access to health services and education significantly challenges the development of children, as well as the development of families and communities, restricting their developmental potential for school and work. The extent to which developmental outcomes of child, family or community is favorable is defined by the realization of basic human rights of access to health and education services. Recognition of the parallel between the development of the child and the development of families and communities has served as the basis for national and global initiatives in health and education in recent decades exemplified by Education for All, the Millennium Development Goals (MDG), and the Sustainable Development Goals (SDG) [1,2]. Implementation of each of these initiatives over the last three decades has been associated with variable progress in addressing the enduring cycles of poverty, infant mortality, malnutrition, illiteracy, and inequalities in access to health care and education of children. However, risks for delay and disability and unmet potential in early development of young children are still pervasive, particularly in low- and middle-income countries [3].

The Declaration of Human Rights in the middle of the 20th century formalized the recognition of basic human rights of access to health and education and provided a standard for referencing the association of inequalities of those rights with limited development of nation states. Recognition of the extent of those inequalities and their disproportionate impact on children and their development was formalized decades later with the United Nations Convention of the Rights of the Child—UNCRC [4]. Although all of the articles of UNCRC define conditions for reducing inequalities and promoting children’s developmental potential, the rights to health and nutrition (Article 24), education (Articles 28 and 29), and articles ensuring that those rights are realized without discrimination of gender, ethnicity, disability and other identities (Articles 2 and 23) are particularly germane to the priorities defined by the MDG and the SDG. This perspective builds on documentation of the status of children, their health and development through comprehensive surveys on national, regional, and global levels. The surveys established the prevalence of child and maternal health conditions and the environmental factors that placed children’s development at significant risk for delay and disability. In higher income countries, documentation of risk factors to children’s health and development led to more effective medical care and improved services for children with chronic conditions. In middle- and lower income countries, such documentation served as the basis for population-based program of disease prevention development of primary care.

Although, documentation of child-environment developmental risk factors has made a great contribution to our understanding of modifiable risk factors, there is a need for a universal framework promoting early child development in low-, middle- and higher-income countries across the health and education sectors, that could guide assessments and interventions as the children grow and develop globally. Therefore, the aims of this paper were to:

(1) Synthesized selected studies identifying children at risk for developmental delay or loss of developmental potential.
(2) Describe key global initiatives to illustrate the impact of environmental factors on children’s developmental potential around the world.
(3) Propose a global framework of child-environment interaction guiding assessment and intervention for health and education.

### Synthesis of Studies and Core Themes of This Paper

Guided by a rights-based approach, this narrative review synthesizes selected studies and global initiatives promoting early child development. Selection for inclusion consisted of the most relevant initiatives to the areas listed in the introduction, decided by consensus, and published in English from 2001 to 2020. Specifically, we synthesized Initiatives addressing environmental factors influencing children’s development, as well as prevalence and estimates of children at risk for developmental delay or loss of developmental potential in low-, middle- and high-income countries. Limitations of this approach are addressed in the paper.

Subsequently, a universal intervention framework of child-environment interactions is proposed for optimizing children’s developmental functioning and progress drawing on the International Classification of Functioning, Disability and Health for Children and Youth (ICF-CY) [5,6]. Finally, we highlight the importance of adopting a global framework guiding assessments and evaluations of children’s development across sectors. As such, the content of this study is organized in three themes as follows:

(a) A global initiative to promote early child development.
(b) A dimensional framework of child functioning and development.
(c) Promotion of children’s development: assessment and intervention in health and education.

## 2. A Global Initiative to Promote Early Child Development

Although initiatives at country levels and programs at global levels by UNICEF, World Health Organization (WHO) and various non-governmental organizations (NGOs) over the last three decades have contributed to significant reductions in the scope and nature of childhood mortality and morbidity, challenges to the healthy development of young children remain pervasive, particularly in low- and middle-income countries, where the largest proportion of children in the world are found. In contrast to earlier population-based studies focusing on specific child or maternal conditions, more recent research is increasingly recognizing the significant role of inadequate or deprived physical and social environments associated with poor developmental outcomes. Increased recognition of the significance of environmental factors is evident in the review by Black et al. [7] of research publications since 2000 indicating that the number of publications on stimulation (*n* = 1121), micronutrients (*n* = 936) and nutrition-related issues (*n* = 508) were 2 to 8 times more frequent than studies of specific conditions such as malaria (*n* = 255), abuse and neglect (*n* = 298) or maternal depression (*n* = 139). This increased research focus on the physical and social environment is consistent with the broader agenda of the MDG and the SDGs. This perspective is in keeping with the challenge of documenting equitable early development as proposed by Barros [2], ”entailing that every girl and boy should have the same opportunities to fully develop their potential, which is only achievable if they have good nutrition, good health, and a rich and stimulating home environment” (p. e873).

Indicators defining the child’s loss of potential and limited opportunities have increasingly become representative of the focus of studies estimating risk for developmental delay, disability or disorders complementing other markers of developmental morbidity. Representative of this perspective is the study by Grantham-McGregor et al. [8] examining developmental potential of children in the context of the first and second MDGs, namely eradicating hunger and poverty and the completion of primary schooling by children globally, respectively. A comprehensive analysis was made of data on children under five in developing countries drawing on the indicators of stunting, available in 126 of these countries, and poverty, available in 88 of the countries. Of the 559 million children under five living in the developing countries, 22% were found to live in poverty, 28% were stunted and 39% were identified as stunted, living in poverty or both. This latter group served as the estimate of disadvantaged children defined in terms of risk for loss of developmental potential. The prevalence estimates for each of the indicators varied widely across regions of the world, with the percent of children living in poverty being lowest in Central and Eastern Europe at 4% and highest in Sub-Saharan Africa at 46%. The range of corresponding values for stunting were 14% for Latin America and the Caribbean and 39% for South Asia. The widest range was found for the combined indicators defining disadvantaged children, with 18% found in Central and Eastern Europe and 61% in Sub-Saharan Africa. The prevalence of lost developmental potential of disadvantaged children was supported by country-specific findings linking stunting and poverty to fewer years of education and limited learning per year of schooling with implications for deficits of income in adulthood.

The estimates of disadvantaged children based on 2004 data by Grantham-McGregor et al. [8] were updated and new estimates were generated for 2010 data in a study by Lu et al. in 2016 [9]. The study used essentially the same approach in the identification of children under five years of age manifesting stunting or exposed to poverty. Recalculating estimates of 2004 data for 141 of the developing countries with improved analytic methods indicated a higher prevalence (51%) of disadvantaged children than the 39% reported in 2007. However, a comparison of estimates based on analysis of data for the 141 countries at two time points, revealed a reduction in the prevalence of disadvantaged children from 51% in 2004 to 43% in 2010. The reduction in the prevalence of children at risk for poor development was attributed largely to reduction in stunting and poverty in South Asia including India and China. Similar to the findings of the Grantham-McGregor et al. [8] study, prevalence estimates varied widely across regions of the world, with the percent of children living in extreme poverty being lowest in Middle East and North Africa at 3% and highest in Sub-Saharan Africa at 54%. The range of corresponding values for stunting were 16% for Latin America and the Caribbean and 47% for South Asia. As in the 2007 study, the widest range was found for the combined indicators defining disadvantaged children, with 21% found in Latin America and the Caribbean and 70% in Sub-Saharan Africa. Lu et al. [9] conclude that findings reflect progress in global efforts to reduce the risk for poor development of young children, but the developmental potential of disadvantaged children continues to be significantly limited in developing countries, particularly in Sub-Saharan Africa. Challenges remain to promote children’s development by reducing stunting and poverty through improved health and increased access to education, reinforcing the premise by Rippoin et al. that “increasing the educational level of their population will lead to better nourished populations, and the ability to improve gross domestic product (GDP)” [10].

The above studies have documented the scope of risk to developmental potential of young children using proxy variables of stunting and living in poverty. In more recent research, surveys such as the UNICEF Multiple Indicator Cluster Survey (MICS) encompass a range of variables more directly linked to developmental aspects of the young child in the Early Child Development Index (ECDI) of relevance in documenting progress to achieving the SDGs. Accessing data from a study of 35 countries, Manu et al. [11] conducted a secondary analysis of literacy-numeracy skills of 100,012 three to five-year-old children. A literacy-numeracy index was derived from the ECDI based on naming 10 letters of the alphabet, knowing four simple words and names and symbols for the numbers 1-10. The index was used to define an outcome variable, classifying children dichotomously as on-track or not on-track. Other variables of interest were availability of children’s books in the home, urban or rural residence, and demographic variables of child age and gender, maternal education and a home wealth index. Analyses revealed that just over half (51.8%) of the children had one book in their home and less than a third (29.9%) met the criteria of being on track for literacy-numeracy. The facilitating role of the home environment was evident in the fact that having a book in the home almost doubled the likelihood of a child meeting the criteria for literacy-numeracy, after adjusting for maternal education, wealth index and demographic variables.

The broader aspects of development as assessed by the ECDI also served as the basis for a study by Gil et al. [12] to document the prevalence of children at developmental risk in low and middle-income countries. In this study, data were available from the administration of the MICS and Demographic and Health Surveys between 2010 to 2016 to families of 330,613 children, ages 3 to 5 years, in 63 low and middle-income countries. In addition to the EDCI, data was also obtained on contextual variables of rural/urban residence, maternal education, child gender and wealth inequality indicators. As in the previous survey studies of children in low and middle-income countries, large variability was found for prevalence of developmental risk between and within world regions as well as between countries. The prevalence of suspected developmental delay by region, based on the ECDI, ranged from a low of 10.1% for Europe and Central Asia and a high of 41.4% for West and Central Africa. Within the West and Central Africa region, the variability ranged from 24.9% for Ghana to 67.3% for Chad. The role of country income on prevalence of suspected developmental delay based on EDCI values yielded parallel findings with a prevalence of 41.2% for low-income countries and only 9.7% for high income countries. Prevalence estimates of suspected delay by assessed domains across all countries was lowest for physical development (3.5%), followed by learning (9.2%) and social-emotional development (24.0%).

Although the issue of promoting early child development as a global initiative is appropriately focused within the framework of inequalities faced by children in low and middle-income countries as illustrated above, initiatives of the SDGs are universal, applying to countries defined by higher incomes as well. Thus, the nature of developmental problems faced by young children, and how those problems are defined and surveyed in highly developed countries do differ, but the focus is the same, that of identifying children at a population level at risk for developmental delay or loss of developmental potential. Estimating the prevalence of children at developmental risk in higher income countries may involve several different surveys as is the case in the U.S. [13]. The National Survey of Children’s Health (NSCH) generates prevalence estimates of 20 specified health conditions, as well as the status of overweight/obesity, risk for developmental delay and having a specified health care need. Based on data of a nationally representative sample of U.S. children 0–17 years of age (N = 91,642), Bethell et al. reported the prevalence of children with a chronic condition to be 43%, an estimate was increased to 54.1% with the inclusion of children with the status of overweight or obesity and risk for developmental delay [14]. Based on screener data to identify children with special health care needs such as needs for medication, additional health, mental health, or educational services, 19.2 % of the children were documented to have special health care needs. The National Health Interview Survey (NHIS) is also administered to nationally representative samples of households with 3–17 year-old children to estimate the prevalence of one of ten diagnosed disabilities (attention deficit hyperactivity disorder [ADHD], autism spectrum disorder [ASD], blindness, cerebral palsy, hearing loss, learning disabilities, intellectual disabilities, seizures, stuttering or stammering and other developmental delay). In a comparative analysis of data from the 2009–2011 to the 2015–2017 administrations of the NHIS, Zablotsky et al. [15] reported an increased prevalence of any diagnosed disability from 16.2% to 17.8%. Among children 3–5 years of age, developmental problems were reflected in prevalence estimates of 10.55% for any disability, 2.73% for stuttering/stammering, 3.30% for learning disabilities, 2.13% for ADHD and 4.67% for other developmental delays.

Reporting on NHIS data for the period 2006 to 2010, Schieve et al. found the prevalence of diagnostic conditions in children 3–17 years of age to be, learning disabilities [LD] (7.8%), ADHD (7.9%), other developmental disabilities [ODD] (4.3%), ASD (0.09) and intellectual disability [ID] (0.07%) [16]. As some children were identified with more than one diagnosed condition, Schieve et al. (2012) derived prevalence on the basis of assignment of children to one of four mutually exclusive groups. This resulted in decreased prevalence estimates as follows, ASD only (0.9%), ID without ASD (0.5%), ADHD without ASD or ID (7.3%), and LD and ODD without ADHD, ASD or ID (5.0%).

As referenced above in the study by Bethell et al. [14], estimates of loss or delay of developmental potential has also been estimated on a functional basis of identifying special health care needs of children. Children with special health care needs were defined by McPherson et al. as children 0–17 years of age “who have or are at increased risk for a chronic physical, developmental, behavioral or emotional condition and who also require health and related services of a type or amount beyond the required of children generally” [17]. Prevalence estimates for children with special health care needs are derived from the inclusion of a screener in the administration of the National Survey of Children with Special Health Care Needs (NS-CSHCN), combined with the National Survey of Children’s Health (NSCH) in 2016. The five screener items are ”(1) need for or use of prescription medications, (2) above-routine use of medical, mental health, or educational services compared with other children, (3) daily activity limitations, (4) need or use of specialized therapies; and (5) need or use of treatment or counseling for emotional developmental or behavioral conditions” [18]. Use of the screener in the NS-CSHCN, the NSCH and the Medical Panels Survey have yielded prevalence estimates of children with special health care needs ranging from 12.8% to 19.3% [13]. A consistent finding of the prevalence estimates derived in these US studies is the role social determinants of poverty and limited parental education, a role shared with risks to development in low and middle-income countries.

## 3. A Dimensional Framework of Child Functioning and Development

Epidemiological studies on the nature and extent of developmental problems of young children globally [3,8,9] reveals that using indicators such as being disadvantaged, at risk for developmental delay and/or experience loss of developmental potential may account for prevalence estimates ranging from 19% to 51% in countries across income levels. Prevalence estimates vary based on the nature of the indicator, from proxy variables of stunting, poverty status, wealth inequalities to caregiver report of the child’s medical condition, health care needs or social and academic skills. Of note is the large variability of children’s risk of poor development between world regions and low and middle-income countries within regions, ranging from 7% to 73% [9]. The role of environmental factors on children’s poor development is evident in the variability of prevalence of risk paralleling variability in the status of economic development of countries. Perhaps the strongest reflection of the role of the home and community environment placing children at developmental risk is high level of suspected developmental delay in literacy-numeracy, across the seven world regions, ranging from 56.3% to 87% [12]. Although the studies utilized different indicators to document developmental risk, the findings provide a consistent picture of problems and delays of development in up to half of all children 3 to 5 years of age growing up in low and middle-income countries. Lower, but comparable prevalence estimates of developmental delay and elevated health care needs in higher income countries supports the premise that developmental risk is a universal concern for young children with implications for policy and practice in the form of SDGs as well as regional and national initiatives.

The gradual decline in the prevalence of children at developmental risk in recent decades with estimates declining from about half to slightly less than 40% of the population in low and middle-income countries [9] has been documented even in the context of variability in how developmental risk was identified. Variability in prevalence estimates may reflect the conceptualization of developmental risk as well as the specific way in how the target problem was defined and cases identified. Different terms defining the population of interest such as “loss of developmental potential “ [8], “disadvantaged children” [9], or “risk for developmental delay” [12] reflect different conceptual and assessment criteria and may thus account for some of the variability of prevalence estimates. However, there is correspondence of these overall prevalence estimates supporting the premise of common, underlying factors of developmental risk. When a more specific definition such as “literacy-numeracy delay” [11] with an associated Literacy-Numeracy Index (LNI) was used in a survey of 35 low and middle-income countries, mean prevalence estimate of children not meeting the criteria for being on-track of LNI was 70.1%, with large variability across countries, ranging from a low of 10.1% to 94.3%.

Although the succession of prevalence estimates over recent decades provides overall documentation of the scope of children at developmental risk, differences in the identification of risk yields different implications for policy and practice. In part, differences in the identification of risk may reflect trends in moving from the use of proxy indicators (e.g., stunting and poverty), subgroup designations (e.g., disadvantaged children) to more specific dimensions of developmental delay (e.g., social-emotional development; literacy-numeracy development). Comprehensive initiatives for children have been listed in term of goals in the MDG and SDG as the basis for defining earlier studies, but an integrated, conceptualization of the developing child within limiting or facilitating environments has been lacking in defining the focus of studies. The importance of a conceptual framework integrating components of child development within a life-course perspective to promote early development of children in low and middle-income countries has been advanced by Black et al. [7]. Central to their approach is the promotion of the child’s developmental potential across the life-course through the provision of nurturing care for health, nutrition, security and safety, responsive caregiving, and early education. Implementation of such an approach recognizes the facilitating role of the systems and policy environment and the caregiving environment of family and community.

Within the ongoing commitment to advance the SDGs for children, a conceptual framework is needed that captures the dimensions of the child’s interaction with the environment defined by the SDG 4 target indicators for early childhood development 4.2, 4.2.1 and 4.2.2. Specifically, the emphasis of the target indicators on children’s access to, and participation in, nurturing and learning environments to ensure their rights to health, learning and well-being requires a conceptual framework integrating dimensions of the child’s developmental interactions with the environment. Central to that framework is a view of early development as the product of the child’s ongoing interactions with the physical, social, and attitudinal environments. The significance of these interactions in defining developmental trajectories and different outcomes of children has been defined in the transactional model of Sameroff and Fiese [19] in which reciprocal child-environment interactions account for developmental change. A similar approach has been proposed by Batorowicz et al. [20] emphasizing transactions as the important feature for interventions for young children. Drawing on the transactional approach, elements of the child-environment interaction need to be defined in dimensional terms of characteristics of the child, of the environment and of the interactions of the child with the environment. To that end, the ICF-CY [4] offers a dimensional taxonomy and accompanying codes of body functions (8 chapters), body structures (8 chapters), environmental factors (5 chapters), and activities and participation (9 chapters), well suited to document the characteristics of the child, the environment and child-environment interactions, respectively.

**Figure 1:**
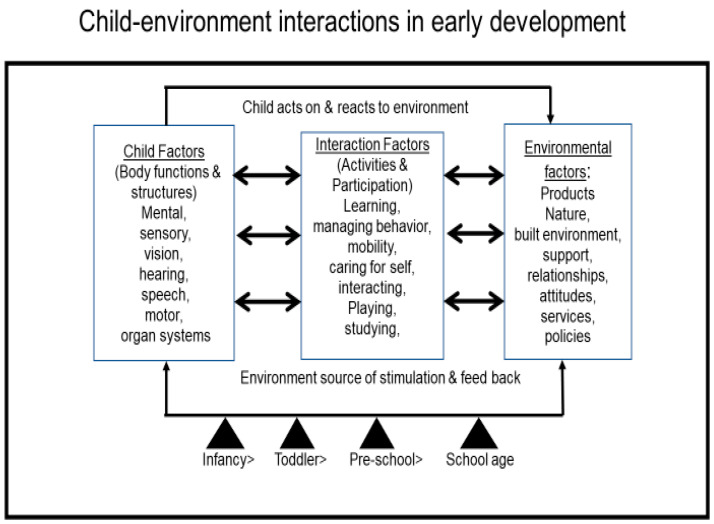
Interactional framework—modeling child-environment interaction within International Classification of Functioning, Disability and Health for Children and Youth (ICF-CY) domains.

Drawing on the findings of the above prevalence studies, the variable or variables defining children’s developmental risk or loss of developmental potential can be identified as elements of the child’s interaction with the environment as shown in Figure 1. Approaching prevalence studies within a framework incorporating the ICF-CY may be useful in several ways [6] in that it promotes focusing on developmental risk in interactional terms rather than solely as characteristics intrinsic to the child. As a universal classification, the ICF-CY codes offer a common language integrating data collection and analysis in health and education sectors that may differ across countries. Further, the codes address the need to assess child, environment and interaction variables in a standard manner across time, for example, in specific contexts such as special education services in Portugal [21] and in broader applications for documenting intervention outcomes in low-and middle-income countries [22].

## 4. Promoting Children’s Development: Assessment and Intervention in Health and Education

Within an interactional framework, assessment, intervention, and evaluation activities should target key developmental indicators, in order to identify and address the specific delays in functioning an individual child may be experiencing. Each of these activities can significantly influence the developmental progress of children who are experiencing delays in development. The timing of assessment and intervention is critical.

Disparities in development can be enhanced or remediated based on the child’s interactions within their environment. Risk factors are barriers and adverse influences on the development of the child. Protective factors are beneficial facilitating development. Exposure to risk and protective factors have cumulative effects [23] that occur when a child is exposed to the same factor repeatedly or different factors with fewer occurrences. Children who have accumulated more risk therefore need more protective factors to promote development. Greater protective factors are associated with better outcomes for children [24]. For example, children who experience multiple risk factors such as poverty, malnutrition, delayed motor development, and delayed speech are impacted by all of the factors cumulatively. Early assessment and intervention practices are key for children facing increased risk, as they allow a mechanism to also increase protective factors during a critical time for child development. A time where gaps in delay can more easily be lessened. Evaluation of assessment and intervention practices allows for more target progress.

Assessment provides information about the level of current functioning, strengths, and deficits, which can then be used to drive the individual goals of an intervention. There are many key issues to consider when planning for developmental assessment. Assessment practices begin with instrument selection. An assessment plan should include gathering baseline information as well as measures to monitor progress. Instrument selection should be culturally relevant, valid, and reliable within the population context. When choosing an instrument, stakeholders should consider the benefits and risks of using a measure.

Although there is a range of developmental assessment measures, particularly in higher income countries, their applicability for use in low-resource countries is limited. Semrud-Clikeman et al. [25] compiled a comprehensive review of neurodevelopmental assessments and screeners for clinicians to determine the best instrument for their goals. In this review, the authors defined the additional considerations needed in order to choose an appropriate assessment tool in the context of low- and middle-income countries. Stakeholders need to determine whether they will use a formal assessment tool, an adapted version of a formal assessment tool, or a locally developed test. Administering a formal assessment tool allows for standardized data to be collected, with scores comparable to a norm reference group. This data provides a clear picture of how an individual child is performing in various domains, it also provides norm referenced scoring which is useful for determining delay. Using formal assessment tools are useful when they are assessing children who are represented in the norm referenced sample.

When assessing children who are in a different cultural context than where the assessment was developed, an approach that has been taken has been to develop an adaptation of the measure for the local context. To avoid overidentification due to cultural bias, and changes in reliability and validity, children should be assessed using data pre- and post- intervention to determine the change in functioning rather than focusing solely on norm referenced data to define delay. Developing local tests can be useful to determine a child’s developmental functioning in the context of their community environment, which may have distinct priorities that shape the developmental trajectory of a child. Though Semrud-Clikeman et al. [25] provide a framework for thinking through the context of a developmental assessment, the bulk of their review provides a matrix of assessments for various neuropsychological domains with specific information about adaptations, training requirements, and test-specific information for practitioners. Their seminal work can be used as a guide for practitioners to begin choosing a specific measure within context of culture, disease, and area of development.

The lack of standardized measures suitable for use in assessment of young children in low-resource countries has been identified by Bhavnani [26] as a significant impediment for population-based screening and for assessing dimensions of child functioning for planning and monitoring interventions. The use of existing tools and measures is often not an option for a number of reasons. From a practical standpoint, the use of standardized, proprietary measures may not be feasible because of cost and the requirement for highly trained professionals to administer them. Lack of standardization with a reference population, concerns about cultural fit, specificity of content and mode of administration are additional constraints on developmental assessment of young children. Recognizing these constraints for valid developmental assessment, particularly of children in rural contexts, a gamified tool was developed to assess cognitive development using tablet technology that could be administered by non-specialists. Specifically, the Developmental Assessment on an E-Platform (DEEP) tool, uses games and narratives on tablets to assess cognitive development of three-year old children across six domains: response inhibition, divided attention, visual form perception, visual integration, reasoning and memory. The cognitive domains were assessed on the basis of the child’s play of nine interactive games such as hidden objects, odd one out, jigsaw and location recall. In a phased testing of alpha and beta versions of the DEEP, the tool generated metrics that reflected individual differences in accuracy and completion of cognitive skills. In a subsequent proof of concept study, Mukherjee et al. [27] administered the DEEP to 200 three-year old children in a rural district in India to test the utility of derived scores to predict children’s performance on a standardized tool, the Bayley Scales of Infant Development-III. Results indicated that DEEP scores were predictive of, and positively correlated with cognitive performance on the BSID-III. The potential utility of the DEEP for developmental assessment of children in low-resource countries is supported by the fact that it yielded reliable data in the context of a developmentally homogeneous study population (mean BSID-III cognitive score of 8), and physical limitations with one-third characterized by stunting and one-fourth being underweight.

Optimal intervention occurs as early as possible, recognizing that disparities in early childhood have long lasting consequences. These consequences can be mediated by early intervention programs in which long-term effects have been noted related to cognitive development, as well as behavioral and emotional development in children [28]. In higher income countries, early intervention focuses increasingly on preparing for educational readiness through programs such as preschool, head start, or home visit interventions. In low- and middle-income countries, additional consequences of health and environmental factors, such as increased risk for malnutrition or infection and disease, may require the creation of different types of intervention feasible in low-resource settings.

Interventions to facilitate improved developmental outcomes for children may involve implementing community-based programs or processes that promote children’s development in low- and middle-income countries. Interventions may target the child directly or aim to involve the interactions between the child and their environment. Eickmann et al. [29] described a community-based intervention program implemented in Brazil in which the intervention program was designed to improve cognitive and motor development by targeting the interaction between the child and their mother. Mothers were trained to implement simple interactions that promote motor and cognitive development within the home environment. Children who received the intervention showed significant improvements in both cognitive and motor development. Children who showed greater initial delays made larger overall gains from the intervention. Children who received the intervention maintained their developmental progress when measured 12–18 months later, while children who did not receive the intervention showed scoring decline.

The intervention included a workshop component as well as home visits. The intervention was designed to increase the mother’s skill through demonstration, practice, skill building, and reinforcement in the home environment. The intervention was intensive, with three workshops and ten home visits occurring over a period of five months. This study highlighted several key factors for optimal intervention, including early assessment leading to targeted intervention for children most at risk. The study also promoted a process that changed the social and environmental interactions of children to increase stimulation.

In upper middle- and high-income countries, intervention priorities may shift toward the developmental risk factors present in the population served to also include school readiness behaviors as part of developmental outcomes. In the U.S., children with developmental delays or other disabilities can begin receiving early intervention services beginning at birth, and eventually provided through the public-school system beginning at age three. Elbaum [30] investigated the developmental outcomes of children with delays (*n* = 17,828) who participated in preschool special education. Children received services to support the development of communication, cognitive, adaptive, motor, and social development. Using the Battelle Developmental Inventory, Second Edition (BDI-2), children were categorized into severity of delay (no delay, mild/moderate delay, and severe delay). Children with a mild or moderate delay (24.2% of the sample, upon entry) made significant gains, with over half (57.6%) exiting the preschool program functioning at age level development. Of the children with a severe delay (60% of sample, upon entry), developmental gains were made as well with 23% exiting the program meeting age level developmental expectations. Children with more complex delays (including delays across multiple measured categories) showed higher level of risk upon entry and less age expected developmental progress upon exiting the program [30]. This outcome speaks to the importance of considering the complex nature of supporting children with multiple areas of delay or children with special healthcare needs, as their developmental needs are unique. These groups of children benefit from early developmental intervention, but may not experience the same immediate gains in developmental progression as children with fewer domain areas of delay or children without special healthcare needs.

Early childhood interventions in Turkey [31] and Austria [32] have structures similar to those in the U.S., beginning at birth with early intervention services which transition to school-based services around 36 months. Early intervention programs in each of these countries are structured by legislation and public policy. They also illustrate similar challenges in cohesiveness across sectors. For example, integrating health and nutrition programs into early intervention services require an integration of intervention from multiple stakeholders. These three countries also face challenges of public awareness of intervention programs as well as limitations with diagnostic requirement for services and lack of opportunities for inclusive programing with typically developing children.

A specific example of an early intervention program implemented in the context of a school-based service, is the Tools of Mind (Tools) program. Recognizing the importance of environmental interactions in intervention, Diamond et al. [33] investigated the impact of Tools on individual and environmental factors in Canadian kindergarten classrooms. The Tools curriculum is designed to teach strategies to support executive functioning skills by teaching contextually based attentional control and self-control strategies. The authors built on previous studies of the Tools intervention to further investigate the impact of the intervention on the environment by measuring the child’s prosocial behavior, academic performance, classroom stress, and teacher burnout. Results of the study, as measured by standardized academic assessment tools and teacher surveys, indicated the contextually based Tools intervention resulted in individual and environmental impacts. Children who were enrolled in a classroom where the teacher used Tools had improved self-regulation skills and performed better academically. Environmental impacts in the classroom included less bullying, increased helping behavior of students, and increased enthusiasm, and decreased burnout among teachers.

In defining replicable strategies of intervention for low-income countries, Engle et al. [34] examined the effectiveness of cross sector programs to optimize the health and the environment of children through intervention. Factors associated with effective intervention included: direct services provide to children, early intervention- especially to those most disadvantaged, and longer or more intensive exposure to the intervention. Additionally, structure and establishing processes were associated with program quality. Though early intervention provides significant benefits strategies to minimize environmental, health, and social risks are also key. This can be challenging as it requires investments beyond direct child intervention, and into other sectors of public policy, research, and governmental programming working together to support comprehensive early child development interventions.

Evaluation of implemented interventions should be considered at both the systemic and individual level. Promoting optimal functioning for children requires the facilitation and coordination of assessment, intervention, and evaluation efforts. Evaluation should include systemic indicators as well as individual indicators. Framed within the lens of transactional theory, Black and Dewy [35] emphasized the importance of integrated interventions, while also magnifying challenges in evaluation. Integrated interventions across health and education target a child’s developmental needs across sectors (i.e., early child development and nutrition). Integrating nutrition programs into early education programs intuitively makes sense, as better nutrition is linked to cognitive development and functioning. Research is needed to demonstrate that nutrition and early child development programs are more effective than early education programs alone [35]. This perspective is elaborated by Lipina and Posner’s [36] comprehensive review demonstrating the relationship of cognitive, linguistic and behavioral development with nutrition and underlying development of brain networks. Within this perspective, interventions, particularly for children in poverty need to address the associated risk factors of malnutrition, inadequate housing, and limited access to health care, support and early education.

Given the importance of integrated interventions to promote children’s developmental potential, meaningful evaluation becomes a key priority. Systematic efforts to evaluate interventions that combine cross-sector programs are challenging in that they require multiple stakeholders to coordinate, organize, and monitor programming. In providing combined health and educational services emphasizing child-environment interactions, there is a need for collaborative initiatives across sectors serving young children, particularly children at risk and with disabilities. Studies focused on child-environment interactions in the educational or health settings. For example, Sanches-Ferreira M et al. [20] described special needs assessment in the Portuguese educational system. Interestingly, the authors’ findings support the need for an expanded focus on person-environment interactions, considering students’ participation in different domains of life—besides learning—as well as the impact of environmental barriers over students’ participation. In addition, they highlight the need for training programs centered on a biopsychosocial understanding of human functioning, the establishment of a transdisciplinary collaborative culture and the use of dynamic assessment tools to equip professionals with appropriate conditions to use the ICF-CY within an interactive perspective. Moreover, Batorowicz et al. [19] proposed a model for research and clinical practice that directs researchers and practitioners working in rehabilitation of young children toward interventions that address the mechanisms of child-environment interaction and that can build capacity within both children and their social environments, including families, peers’ groups and communities. In this service provision approach, the authors highlight that health is created and lived by people within the settings of their everyday life; where they learn, work, play, and love [19].

Table 1 shows the key findings of the studies included in this section applying a child-environment interaction framework. Promoting early child development, we have identified children’s factors, environmental factors, and interaction factors facilitating child development and functioning in different settings (Table 1).

## 5. Contribution to the Field of Early Child Development

This narrative review highlights the pivotal studies supporting the role of environmental factors and interaction factors influencing child development, developmental potential, and functioning trajectories globally (Table 1). In addition, the selected studies show the gaps related to the lack of comprehensive and collaborative frameworks of assessment and service provision in early child development across sectors. Hence, guided by the ICF-CY, we propose an intervention framework of child-environment interactions to optimize children’s developmental functioning and trajectories at a population level (Figure 1). The proposed interactional framework can guide the identification of key developmental indicators for assessments, interventions, and evaluations. Moreover, the universal adoption of the ICF-CY and pediatric ICF-based tools as guiding frameworks for comprehensive assessment of children’s development and optimal functioning across health and education, promotes professional training on integrated assessments, evaluations, and interventions, fosters collaborative service provision across sectors, and facilitate communication among multiple stakeholders [6,37,38,39]. Systematic efforts to use a common language in assessments and interventions across sectors will facilitate continuity of care, coordination, organization, and monitoring of programs throughout the children’s developmental trajectories.

### Limitations

Despite the wide scope of this narrative review of global initiatives reporting on children, families and environmental factors impacting early child development, it is important to note some limitations. The main limitation relates to the authors’ personal preferences and areas of expertise which could have influenced the selection of studies reviewed in this paper. Moreover, we included studies published in English, therefore, we might have missed important information published in other languages.

## 6. Conclusions

The goals of assessment to promote children’s developmental trajectories are to gather accurate data about the child’s level of functioning across domains of cognitive, communicative, motor, social and adaptive development. Such assessment provides the basis for identifying areas of strength and if delays exist- while also considering the cultural context of the child and communicating the assessment findings to specify targeted intervention. Given the significant need, but limited availability of developmental measures appropriates for low and middle-income countries, an important priority in the continuing implementation of SDG is the development of measures that assess proximal aspects of child functioning that can complete more distal, proxy measures of development such as poverty and stunting. The ECDI and the literacy-numeracy index, and the development of the DEEP represent measures appropriate for assessing developmental functioning of young children in low-resource countries. The integration of multiple sectors, although challenging yields potential benefits to the evaluation process. To this end, Black and Dewey recommend designing evaluative processes with multiple integrated interventions in mind, examining population factors, and then promoting capacity among care providers to support evaluation from a theory-based perspective [35].

This narrative review advances the importance of adopting a universal child-environment interaction framework in initiatives to promote the early development of children around the world. Using the ICF-CY as a guiding framework, assessments and interventions can be planned and delivered considering dynamic child-environment interactions, facilitating communication across sectors, and facilitating continuity of care in the changing environment—home, school, community—where children grow, develop, and play. 

### Future Direction

For children to realize their developmental potential, there is a priority to ensure equitable access to appropriate screening, assessment, and intervention. As countries vary in terms of environmental, societal, and economic challenges, it is important to consider country contexts when developing and evaluating assessment and intervention initiatives. Considering low cost and sustainable options for assessment and intervention can reduce financial barriers. Incorporating technology into early intervention and assessment practices can decrease barriers imposed by distance and increase access to trained professionals. Incorporating mechanisms for identifying culturally appropriate assessment practices increases accuracy of identification of need while reducing the potential for overidentification of delays. Capitalizing on the expertise of interdisciplinary teams can also support stakeholders across sectors to ensure continuity and proper administration of interventions. Adoption of a common language to report outcomes can facilitate comparison of outcomes and interventions across sectors, programs, and countries. Continued work is needed globally to support children’s developmental trajectories toward positive outcomes. This is especially true as countries adapt assessment and intervention practices in the face of evolving environmental impacts of poverty, natural disasters, pandemics, societal and economic changes to prevent the loss of developmental potential of all children.

## Figures and Tables

**Table 1:** Promoting early child development: a child-environment interaction framework.

Study Authors	Child Factors	Interaction Factors	Environmental Factors
**Eickmann et al. 2003**	Cognitive functions Motor functions	Increase play interactions between mother and child Increase maternal knowledge of development, while teaching skills Changing of maternal self-perceptions	Home environment factors Addressing limited resources Early assessment and intervention Skill reinforcement in home visits
**Elbaum, 2020**	Communication Cognitive functions Motor functions Psycho-social functions	Promoting and supporting developmental progress Increase ability to participate in activities typical for same aged peers	Provision of preschool special education services under Part B-619- IDEA legislation Children deemed eligible for special education services through federal guidelines
**Engle et al. 2007**	Sensorimotor functions Cognitive functions language development Psycho-social functions Physical functions	Parenting/parent-child interactions Provision of nutrition and education) Early, intense intervention programing Enhanced, coordinated intervention Families partnering in intervention	Center based programing Optimal environment: early childhood programs with structure and established processes Environmental risks- toxins Infectious risks: HIV/AIDS, malaria Social risks- maternal depression, experiencing violence, abuse, or neglect
**Diamond et al. 2019**	Cognitive functions Executive function) Psycho-social functions	Teacher/Child interactions Peer interactions Play based intervention environmentally embedded in kindergarten classroom	School based programming, embedded in kindergarten curriculum Protective factors- proactively teaching executive functioning skills in generalizable context Addressing social risks- bullying, loneliness
**Black and Dewey, 2014**	Cognitive functions Executive function) Psycho-social functions	Learning through enrichment experiences Responsive interactions with caregivers Providing structure and encouragement	Addressing barriers associated with poverty Integrated interventions that include nutrition and early child development
**Semrud-Clikeman, 2017**	Neurodevelopmental functions Attention/memory functions Visual-motor, and motor functions	Provision of appropriate developmental assessment within the country of interest Child’s experiences and world view	Assessment in language of origin Context of assessment administration Assessment tasks matching culturally developed abilities
**Mukherjee et al. 2020**	Cognitive functions Physical functions	Cognitive assessment of development of 3-year old children with gamified measure Performance predictive of standardize measure of development	Low-cost technology Tablet with games Utility in rural context

## Data Availability

Data sharing is not applicable to this article as no new data were created in this study. The data supporting our proposed framework are available within the article.
